# Self-reported pain and disability outcomes from an endogenous model of muscular back pain

**DOI:** 10.1186/1471-2474-12-35

**Published:** 2011-02-02

**Authors:** Mark D Bishop, Maggie E Horn, Steven Z George, Michael E Robinson

**Affiliations:** 1 Department of Physical Therapy, University of Florida, Gainesville, Florida, USA; 2 Rehabilitation Doctoral Program, College of Public Health and Health Professions, University of Florida, Gainesville, Florida, USA; 3 Center for Pain and Behavioral Health, University of Florida, Gainesville, Florida, USA; 4 Department of Clinical and Health Psychology, University of Florida, Gainesville, Florida, USA

## Abstract

**Background:**

Our purpose was to develop an induced musculoskeletal pain model of acute low back pain and examine the relationship among pain, disability and fear in this model.

**Methods:**

Delayed onset muscle soreness was induced in 52 healthy volunteers (23 women, 17 men; average age 22.4 years; average BMI 24.3) using fatiguing trunk extension exercise. Measures of pain intensity, unpleasantness, and location, and disability, were tracked for one week after exercise.

**Results:**

Pain intensity ranged from 0 to 68 with 57.5% of participants reporting peak pain at 24 hours and 32.5% reporting this at 48 hours. The majority of participants reported pain in the low back with 33% also reporting pain in the legs. The ratio of unpleasantness to intensity indicated that the sensation was considered more unpleasant than intense. Statistical differences were noted in levels of reported disability between participants with and without leg pain.

Pain intensity at 24 hours was correlated with pain unpleasantness, pain area and disability. Also, fear of pain was associated with pain intensity and unpleasantness. Disability was predicted by sex, presence of leg pain, and pain intensity; however, the largest amount of variance was explained by pain intensity (27% of a total 40%). The second model, predicting pain intensity only included fear of pain and explained less than 10% of the variance in pain intensity.

**Conclusions:**

Our results demonstrate a significant association between pain and disability in this model in young adults. However, the model is most applicable to patients with lower levels of pain and disability. Future work should include older adults to improve the external validity of this model.

## Background

Musculoskeletal pain is the most common form of chronic or recurrent pain [1,2] that has a high societal cost [3], making it a public health priority [1,2,4].

Current literature suggests that there is a potentially complex interaction of factors that contribute to low back pain (LBP). Psychological factors, for example, prolong recovery and may predict disability for patients with LBP [5]. One limitation to clinical studies is that there can be little experimental control of the pain experience. Using a model of experimentally induced pain, the type of the pain stimulus, as well as the general area where pain is experienced can be controlled [6].

Exercise, an endogenous method of inducing muscle pain, can produce pain during [7] and after activity [8]. Performance of eccentric (lengthening) muscle actions in muscles unaccustomed to such forces causes damage to muscle fibers. Pain, hyperalgesia, allodynia, edema, and weakness can also result [9-13]. These symptoms and signs typically completely resolved within two weeks [14,15] and are referred to as delayed onset muscle soreness (DOMS). DOMS has been reported in the trunk muscles after floorball training [16] and performance of the tests associated with a functional capacity evaluation [17,18]. However, each of these models involved significant time to induce the DOMS. For example, participants in the study by Hjortskov et al, played handball for two hours while participants in work described by Soer et al completed a functional capacity examination, a procedure that involves twelve separate strength tasks.

In DOMS models that involve peripheral (non-axial) muscles common protocols target a primary muscle or muscle group in which the DOMS is to be induced. Active trunk exercises have been used to generate DOMS in the trunk [19,20]. Therefore we had two general goals to extend previous work in this area. Our first goal was to describe the pain and disability outcomes resulting from DOMS to establish the external validity of DOMS as a model of LBP. We aimed to quantify pain intensity and self-report of disability, examine the distribution of any pain that might occur expecting that it would be in anatomic regions consistent with LBP reported by patients seeking intervention for their pain, and characterize the quality of any pain reported. Additionally we sought to identify whether peripheral sensitization occurred following DOMS in the trunk, as indicated by reductions in mechanical pain thresholds over the targeted muscles.

Our next goal was to determine the relevance of this model to patients with low back pain. We hypothesized that there would be a relationship between pain intensity and self-report of disability [21-23] and we also expected variability in pain intensity to be related to psychological factors, such as pain related fear, and psychophysical measures [24,25]. Then we compared pain and disability reports by participants in the current study to a group of age-matched patients seeking interventions for LBP. Demographics and the characteristics of the patients' pain experiences were tracked as part of a separate randomized trial of interventions for LBP [26]. We hypothesized that there would be overlap between the two groups (healthy participants with DOMS and patients with LBP) in reports of pain intensity. The overriding rationale for these hypotheses was that if measures collected after DOMS were consistent with observations made of patients seeking intervention for LBP this would support the external validity of the model.

## Methods

### Participants

52 healthy pain-free volunteers (23 women, 17 men; average age 22.4 years; average BMI 24.3) read and signed an informed consent form approved by the University Institutional Review Board. Participants were excluded if they met any of the following criteria: previous participation in a conditioning program specific to trunk extensors, any current back pain, any chronic medical conditions that may affect pain perception, kidney dysfunction, major psychiatric disorder, history of previous injury including surgery to the lumbar spine, cardiac conditions, osteoporosis, or liver dysfunction, or performance of any intervention for symptoms induced by exercise before the termination of their participation in the protocol.

### Measures

All tests and measures were collected in a research laboratory setting before exercise and at 24, 48, 96 hours and one week after the exercise protocol was administered. In addition, participants complete pain and disability questionnaires 2 weeks, 4 weeks and 12 weeks after exercise to examine any long term effects of participation.

#### Pain intensity, unpleasantness, and location

Pain intensity was measured with a visual analog scale (VAS) consisting of a 100 mm line anchored at one end with "none" and at the other with "'worst imaginable." A previous study has indicated that the VAS is a valid ratio measure [27]. Participants rated "worst pain in the back or legs today" by placing a mark along the 100 mm line. The VAS for pain unpleasantness consisted of a 100 mm line with anchors of "not at all unpleasant" and "most unpleasant imaginable." Pain intensity measures the sensory-discriminative dimension of pain, and pain unpleasantness measures the affective-cognitive dimension of pain [28]. The affective ratio (unpleasantness divided by intensity) provides information about the quality of the pain [29]. Clinical pain often is associated with unpleasantness being similar to, or greater than, intensity [30]. Participants also completed pain drawings to indicate the spatial distribution of any back or leg pain. Data were coded to indicate if the participant reported any leg pain defined in this study as pain below the gluteal fold.

#### Disability

Self-report of low back-related disability was assessed with a modified version of the Oswestry Disability Questionnaire (ODQ) [31]. This ten-item questionnaire has a range of 0 (no disability due to back pain) to 100 (completely disabled due to back pain) and is typically reported in percentages. The ODQ has been extensively used as a measure of disability in those patients with LBP during clinical management and experimental studies. The modified ODQ has very good test-retest repeatability [31] and a recent consensus statement included the ODQ as an important measure of physical functioning in patients with LBP [32].

#### Pain-related fear

Two questionnaires were used to measure pain-related fear. The Fear of Pain Questionnaire (FPQ) is a 30-item, 5-point rating scale with a range from 30 to 150, developed to measure fear about specific situations that may cause pain. We used the total score for the FPQ, as we were most interested in measuring participants' general fear of pain [33]. High levels of internal consistency (>0.8) have been reported in clinical and non-clinical groups of participants [33,34]. This questionnaire was used because it can be completed by healthy, pain free participants and we have found it to be associated with experimental pain responses in our previous studies [24,25].

A shortened version of the Tampa Scale of Kinesiophobia (TSK-11) was used to assess the fear of movement or injury [35]. The original TSK included 17 questions and was used in multiple studies in participants with low back pain [36-38]. The TSK-11 provides a measure of 2 separate domains: somatic focus and fear of reinjury [39]. The TSK-11 excludes 6 questions from the original Tampa Scale of Kinesiophobia (TSK). The TSK-11 has demonstrated similar factor structure, reliability (ICC = 0.82), and validity to the original version of the TSK [35]. The items are scored on a 4-point scale from 1 (strongly disagree) to 4 (strongly agree). Lower scores on the TSK-11 indicate less pain-related fear of movement.

#### Pain Sensitivity

Mechanical Pressure Threshold (MPT): Lowered mechanical pain threshold has been previously reported in DOMS models suggesting sensitization of muscle nociceptors in response to mediators of the inflammatory process [6,40].Tenderness in paraspinal muscle tissue was assessed using a hand-held dynamometer (Microfet 2, Hoggan Health Industries, Inc, West Jordan, UT). The tip of the dynamometer is equipped with a rubber foot-plate of 1-cm diameter. During testing, the participant was positioned in prone and force was applied until the participant reported that the sensation changed from pressure to pain. At that point the participant rated pain they experienced using a numeric rating scale (NRS) anchored at 0 (no pain sensation at all) and 100 (worst pain imaginable) and the applied force to reach this threshold was recorded in kg-force. Threshold measures were evoked in the paraspinal muscles bilaterally 2.5 cm from the spinous processes of L1, L5 and S2 for a total of six ratings. These were averaged to provide a single measure of MPT. Between session reliability for MPT has been shown to be high (ICC > 0.87) in the posterior trunk muscles [41].

### Exercise Protocol

Prior to exercise all participants completed a submaximal effort warm-up session consisting of riding the stationary bicycle at a speed of 50-60 RPM and 1 Kp of resistance and static passive stretching (held for 30 seconds) of the lower extremities and posterior trunk. Participants performed an isometric (static) test of total torque of the trunk extensor muscles through their available trunk flexion range of motion (ROM) using a MedX lumbar extension exercise machine following the standardized protocol [42]. The repeatability of isometric torque production is well-established in participants without pain [42] and groups of patients with LBP [43]. Participants were seated in the MedX machine with the stabilizing straps attached across the pelvis and knees. The participant was moved through the ROM of the machine in lumbar flexion and extension to determine his or her available ROM. The device was locked into place in maximal flexion and the participant was instructed to build up force gradually against a pad in contact with the mid and lower back. The torque generated by the participant was displayed graphically on the data collection computer. Once peak effort was observed by the research assistant, the participant was instructed to relax, the device released and the participant returned to an upright position for at least 10 seconds. Isometric testing was administered seven times in positions that ranged from the participant's maximum available trunk extension to maximal trunk flexion. The isometric torque collected at each test angle was summed to give measure of total torque produced across the entire range of motion.

After baseline total torque was recorded, participants performed bouts of dynamic exercise to the point of volitional fatigue. To perform the exercise bout, the participants were seated and restrained in a MedX lumbar extension exercise machine. Participants performed as many repetitions as possible using a weight load equal to approximately 80% of the peak torque measured during the isometric test. Each repetition was performed through the full available ROM and the participants were encouraged to perform the lifting portion (concentric) in two seconds and the lowering (eccentric) in four seconds. Repetitions continued until the patient reported being unable to move through a full range of motion (volitional fatigue). Once this occurred, the isometric torque test was performed again. If the total torque measured during the repeat isometric test was 50% or less of the baseline total torque, the protocol was complete. If this didn't occur, the exercise bout was repeated. Participants repeated this sequence of dynamic exercise and isometric testing until total measured torque decreased to 50% of the baseline measurement. Participants were instructed not to initiate any medication in the next 48 hours, or apply any intervention, such as ice or heating pacs to the lumbar spine.

### Analysis

Descriptive statistics were generated for all baseline variables.

#### Effect of DOMS

The effect of exercise on pain intensity and disability was compared over time using separate repeated measures analysis of variance (ANOVA) models. We specifically expected pain reports to peak at 24 or 48 hours and return to baseline over time. Additionally, we calculated the affective ratio to examine the change in quality of the pain. These ratios represent the ratio of unpleasantness to intensity and were calculated beginning at 24 hours. To examine changes in pain sensitivity that occurred in response to DOMS we used a repeated-measures ANOVA. We also compared participants with and without leg pain on measures of pain, disability and fear.

Changes in muscle performance were also examined. A two-way repeated measures ANOVA was used to examine whether decrements in isometric torque production were specific to a particular angle or angles, or whether changes occurred across all testing angles equally. Additionally the total isometric torque was tracked to determine the timeframe over which muscle performance returned to baseline levels of performance.

#### Model Validity

Next we examined the extent to which pain reports explained self-reported disability after the induction of pain. We used the values of pain and disability at 24 hours following the induction of muscle pain for these analyses. First, zero order correlations were calculated among the potential predictor and outcome variables. Sex differences in pain intensity and disability were tested using independent t-tests. The first model was fit for disability using the ODQ as the dependent variable. We built a hierarchical regression model using demographic variables as the first block. The second block consisted of psychological measures for which the zero order correlation with pain was significant at p < 0.1. Pain intensity was entered as the final block. Hierarchical regression was chosen to allow us to examine the contributions of each 'block' to the overall variation in the dependent variable. We examined the change in R^2 ^and standardized betas to determine significant contributions to the variance.

A second model was build to examine factors that explained variability in pain intensity after exercise. The first block entered consisted of demographic variables. The second block consisted of psychological measures for which the zero order correlation with pain was significant at p < 0.1.

Our last test of validity was to compare the experimentally induced LBP to clinical LBP. Reported pain intensity from participants in our study was compared to age matched patients with low back pain from a previous clinical trial [26] using Mann-Whitney U test. Demographic details of patients in the clinical trial are summarized in Table 1.

**Table 1:** Characteristics of participants performing the exercise protocol and age matched-patients with LBP.

	Healthy participants (n = 52)		**Patients with LBP**[26]** (n = 33)**	
Variable	Median	Range	Median	Range
Age (years)	23	18, 32	24	18, 32
Sex (% female)	31	60%	22	66%
Race/Ethnicity				
White	42	81%	25	76%
African American	5	10%	5	15%
Asian	4	8%	1	3%
Native American	0	0%	0	0%
Hawaiian/Pacific Islander	1	2%	1	3%
More than one race	8		-	
Pain intensity	16	0, 63	50	0, 70
Disability	4	0, 30	28	0, 74
Fear of pain (FPQ-III)	74.7	30, 110	-	
Kinesiophobia (TSK)	18.5	11, 26	-	

Repeated measures ANOVAs were performed using StatView 5.0.1 (SAS Institute, Cary, NC, USA), and regression analyses were performed using SPSS for Windows 15.1. Type 1 error was maintained at 5%.

## Results

Demographic and descriptive data are summarized in Table 1. 52 participants completed the evaluations without drop-outs and no adverse events were reported. 38 participants indicated that they had not performed any type of regular exercise (more than one a week) for the past month. Five indicated participating in weight-training (no training of the trunk extensors), and seven indicated that they performed aerobic exercise (running, walking, or bicycling) twice a week.

### Effects of DOMS

**Figure 1:**
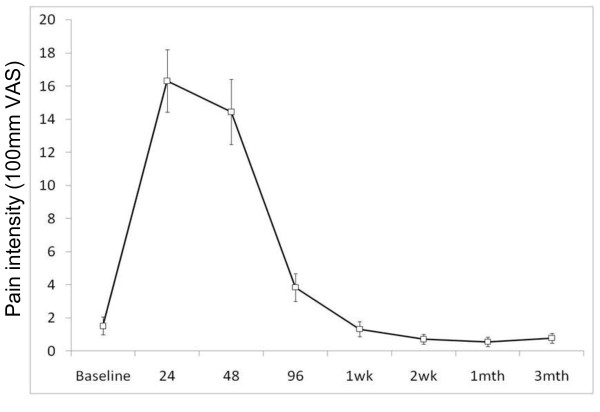
**Pain intensity reported after induction of DOMS**.

**Figure 2:**
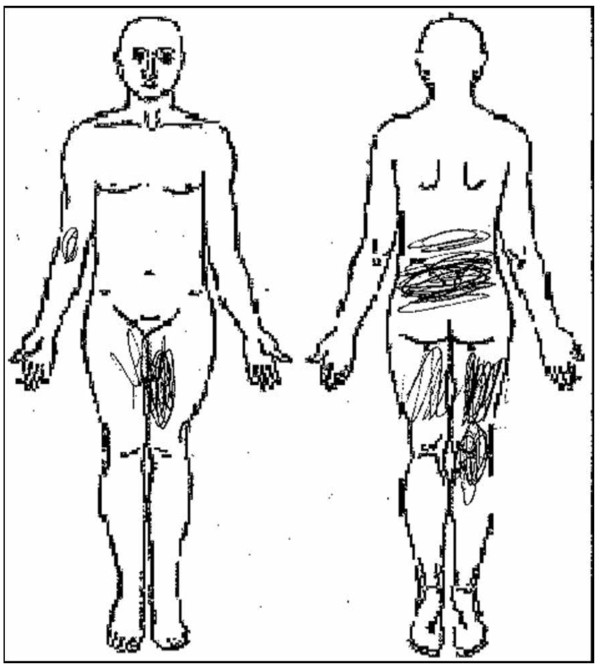
**Sample pain diagram of subject reporting a "peak pain in the back or legs" of 30 mm at 48 hours**. Participants were instructed to "use the diagram to mark the areas in which you are feeling pain as a result of the exercises done in this study".

There was a significant main effect of time for pain intensity in the back or legs (F_7,58 _= 45.7, p < 0.001). The general time course of DOMS is shown in Figure 1. 30 participants reporting peak pain at 24 hours and 17 reporting this at 48 hours. One participant reported peak pain at 96 hours after exercise and 4 participants reported no pain at any time period.18 participants reported pain in the back and legs. No participants reported pain below the knee at any time point. A sample pain diagram is shown in Figure 2 illustrating the distribution of pain reported by this participant at 48 hours. The pain intensity for 45 of participants had returned to zero within one week after the induction of DOMS and only 1 participant reported pain at 2 weeks (10 mm). Similarly, the distribution of disability scores increased from baseline to 24 hours and then returned to zero over time (F_7,58 _= 13.2, p < 0.001). MPT at 24 and 48 hrs was significantly lower than baseline (p = 0.004 and p = 0.012, respectively) and returned to baseline by 96 hours. These values are shown Table 2.

**Table 2:** Changes in Mechanical Pain Threshold

	Force (kgf)	Rating (NRS)
Baseline	28.8 ± 1.4	23.1 ± 2.3
24 hours	23.6 ± 1.3	26.9 ± 2.6
48 hours	24.8 ± 1.2	24.1 ± 2.4
96 hours	27.7 ± 1.2	22.7 ± 2.2

We assessed the affective ratio over the course of one-week. The affective ratio did not follow the same pattern as pain intensity and disability; however there were differences across testing sessions (F_1,51 _= 8.81, p = 0.004). The ratio of unpleasantness to intensity statistically increased from 24 to 48 hours (p = 0.010) and remained elevated one week after the exercise protocol (p = 0.007) following a different time course from measures of intensity only. The ratio of unpleasantness to intensity remained greater than one indicating that the sensation was considered more unpleasant than intense.

**Figure 3:**
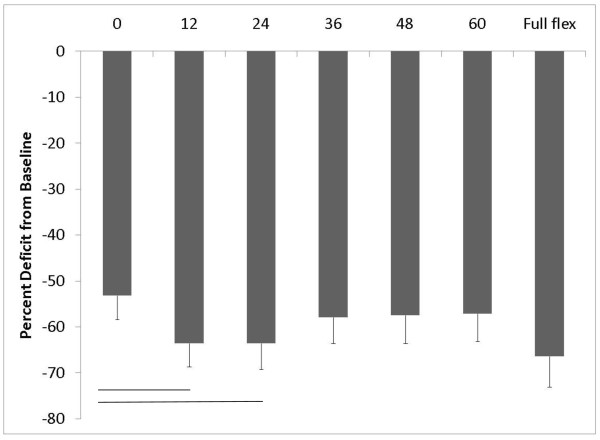
**Decrements in isometric torque production immediately following the exercise protocol**. The line represents differences among testing angles (p < 0.002 after Bonferroni correction).

**Figure 4:**
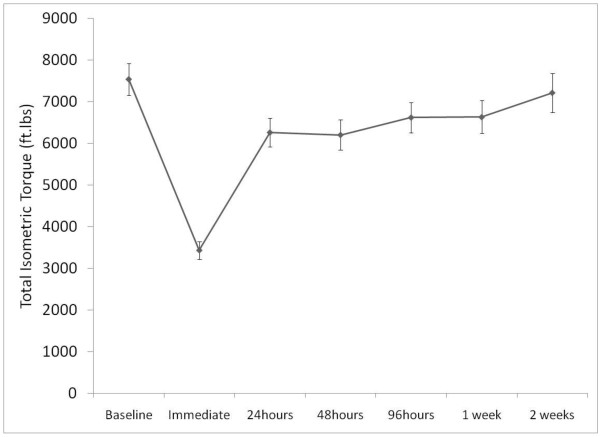
**Time course to recovery of isometric torque production in the trunk extensor muscles**.

When immediate changes in muscle performance after exercise were examined, a significant interaction was noted between time and angle (F_6,312 _= 15.01, p < 0.001). The greatest decrements in isometric torque production occurred at test angles 12, 24 and 72 degrees corresponding to the mid-range of trunk motion and in full trunk flexion, respectively. Post-hoc testing indicated that the decrement in torque at 12 degrees and at 24 degrees was largerer than in the loss of torque in full extension. The percent deficit at each angle is shown in Figure 3. Total isometric torque remained depressed at one week after the exercise protocol was performed and returned to baseline totals by two weeks after exercise (Figure 4).

### Model validity

The pain intensity at 24 hours for the participants ranged from 0 to 63 on the VAS. Statistical differences were noted in levels of self-reported disability when comparing participants with and without leg pain (participants reporting leg pain had higher levels of self-reported disability) but no differences were noted in pain intensity.

The zero order correlations among demographic, psychological, and pain variables, and self-reports of disability at 24 hours are summarized in Table 3. Pain intensity at 24 hours was correlated with pain unpleasantness, pain area and self-reports of disability. Also, fear of pain was associated with pain intensity and pain unpleasantness.

**Table 3:** Bivariate associations among measures

	Pain Unpleasantness	Pain Area	Disability	Age	BMI	TSK	FPQ
Pain intensity	0.53**	0.33**	0.56**	-0.05	0.14	0.06	0.26*
Pain Unpleasantness		0.03	0.31*	-0.10	0.07	0.17	0.35**
Pain Area			0.23	-0.08	0.01	0.05	<0.01
Disability				-0.11	0.05	0.10	0.20
Age					0.11	-0.02	-0.11
BMI						-0.15	0.03
TSK							0.49**

Disability: Oswestry Disability Questionnaire

BMI: Body Mass Index

TSK: Tampa Scale of Kinesiophobia

FPQ: Fear of Pain

*- p < 0.05, **-p < 0.01

Our first model, predicting self-report of disability at 24 hours, included sex, as the only demographic variable, the presence of leg pain, and pain intensity. Each variable contributed unique amounts of variance to the final model predicting disability as indicated by the significant changes in the adjusted R^2 ^(see Table 4). However, the largest amount of variance in disability, an additional 27%, was explained by pain intensity after controlling for sex and the presence of leg pain. The final model accounted for 40% of the total variance in self-reported disability and is shown in Table 4.

**Table 4:** Model Predicting Self-report of Disability

Step		Beta	Sig.	**Adjusted R**^2^	Sig. F Change
1	(Constant)	2.08	0.060	0.06	0.035
	Sex	3.03	0.035		

2	(Constant)	0.61	0.610	0.14	0.015
	Sex	3.52	0.012		
	Leg pain	3.53	0.015		

3	(Constant)	-2.31	0.047	0.41	<0.001
	Sex	3.36	0.004		
	Leg pain	2.85	0.018		
	Pain intensity	0.20	<0.001		

No other predictor variables of interest were related to pain-intensity except fear of pain; therefore no regression analyses were performed. The association between fear of pain and pain intensity is shown in Table 3.

Thirty-four patients in the comparison clinical trial [26] were between the ages of 18 and 32 years. Demographic details of this group of patients is presented in Table 1. Data from these patients was compared to the reports of pain from participants in this DOMS study. Overall, the reports of pain after exercise were significantly lower than the reports of pain of the patients (U = 256.5, p < 0.001). Visual inspection of the quartile ranges in both data sets suggested that the highest pain intensity reports of the experimental group (50^th ^percentile and greater) overlapped with the lowest pain reports (50^th ^percentile or lower) of the patients seeking intervention for their back pain - 16 to 68 mm, and 0 to 50 mm respectively.

## Discussion

We induced acute pain using an exercise protocol to create DOMS in the low back and followed participants for 12 weeks to collect pertinent outcomes. DOMS models are potentially very useful because they mimic musculoskeletal pain in loss of range of motion, pain with movement and self care behaviors [12,13,44]. The reports of pain generated in this current study followed a time course consistent with other DOMS models with pain peaking at 24 or 48 hours and resolving within approximately one week after exercise [45,46].

The reported pain intensity ranged from 0 to 68 mm in the low back and, sometimes, legs. These anatomic areas are consistent with regions described by patients seeking intervention for LBP. In patients with low back and leg pain, the leg pain may be referred from anatomic structures in the lumbar spine. For example, structures that have been demonstrated to cause referral of pain into the posterior thigh include the dura and nerve roots [47] as well as the intraspinous ligament [48] and the lumbar multifidii [49,50]. Magnetic resonance imaging of the posterior trunk muscles after performing the exercise protocol used in this manuscript indicates that primary changes identified after exercise occur in the lumbar multifidii (paper in review). The leg pain in our current study may also have resulted from the sustained isometric contractions performed by muscles of the lower extremity during the exercise protocol. While these muscles were not subjected to the forces that occurring during eccentric (lengthening) actions, there is evidence to suggest that restricted blood flow to a working muscle may cause DOMS in that muscle [51].

However, this range of reported pain intensity is lower than that reported by Udermann et al, 2002 who also used trunk extension exercise to cause DOMS. These authors present data indicating that five participants who completed 50 repetitions of trunk extension using a weight that was 100% of the isometric maximum reported pain intensity of approximately 8 using a 10 cm VAS that peaked at 24 hours after exercise. This exercise intensity was somewhat greater than we used in our model although no information regarding anchors on the VAS or confidence intervals reported, and no error bars are shown in the graph. When developing the model used in the current study we were very cautious about the magnitude of the loading being placed on the lumbar spines of the participants. This was a concern of the funding agency and of our own institutional review board. Consequently, our protocol is less aggressive than is seen in models developed in the extremities and included both concentric and eccentric muscle actions. Additionally participants in our current study were asked to rate pain that they were experiencing at rest, not during movement or activity. These factors combined may explain why the pain intensity reported is less than other models of DOMS.

Consistent with other DOMS models, participants experienced an increase in the affective-cognitive component of the pain experience (unpleasantness) [44,52]. The affective ratio showed a slightly different pattern of change over time. At each time point the ratio was greater than one indicating that the pain sensation was perceived as more unpleasant than intense. This also supports model clinical relevance because clinical pain often is associated with unpleasantness being similar to, or greater than, intensity. The ratio increased at 48 hours and remained so at the one week follow-up. By this time, pain intensity had decreased to baseline for most participants. Fields et al, 1999 has suggested that for a sensation to be recognized as painful there must be unpleasantness associated with the sensation intensity. We did not specifically query participants about the quality of any ongoing sensation (other than unpleasantness) that they may have been feeling in their back and legs but we speculate that participants may have continued to experience 'soreness' but not pain per se.

Additionally, participants reported disability after induction of DOMS using an outcome measure used in clinical studies of patients with LBP. In our study, pain intensity was the primary predictor of this self-report of disability. This is relationship is also consistent with clinical populations. For example, in nurses with back pain this correlation was 0.69 [21], compared to r = 0.50 in our study. The presence of leg pain was also associated with disability in our model of LBP and this finding is likewise consistent with the clinical literature regarding LBP. Selim et al, 1998 compared groups of patients with LBP who were then further classified by the location of leg pain (none, to the knee, below the knee). These authors reported that disability increased as the involvement of leg pain increased [53].

However, the range of scores using the self-report of disability reported in our study was 0 to 30. This range suggests that, while participants perceived some disability from DOMS, it was a minimal amount of disability. This is not unexpected given that participants are told during the informed consent process to expect the pain that they experience to be of short duration and instructional set regarding what to expect after a procedure has been demonstrated to influence the outcome after that procedure [54]. However, this is also a strength of the model as there were a range of pain intensity values generated and it may be unethical to have induced models that cause high disability conditions. Such a model - long duration of high pain and disability - would no longer be a model per se but rather the clinical condition being modeled!

Lowered mechanical pain threshold has been reported in DOMS models involving differing somatic locations such as the biceps [29], shoulder [55], and hand [10]. A proposed mechanism of DOMS is that muscle nociceptors become sensitized in response to mediators of the inflammatory process, thereby lowering stimulus thresholds (peripheral sensitization) [6,40]. The finding of local reduction in mechanical pain threshold after DOMS was consistent with other studies of DOMS [22,56]. In addition, isometric torque producing ability of the trunk muscles remained impaired for approximately 2 weeks after exercise. These findings in combination confirm that the exercise protocol produced muscle changes in the target muscles.

Pain intensity in our model was only related to baseline fear of pain and not to kinesiophobia as measured by the TSK. The most likely explanation for this finding is the type of participants in our study. The version of the TSK used in our study was TKS-11. This questionnaire includes questions about the current pain experienced by the individual completing the form. All our volunteers were pain-free and none had experienced back pain or had an accident. More recently the TSK-general has been published. This may have been a more appropriate measure of kinesiophobia in our participants. In contrast, the FPQ measures general fear of pain and may represent a trait measure of fear.

Our findings are also different from those of Soer et al, 2009. These authors report that sex was a significant predictor of pain intensity in their model of DOMS after an FCE. For our study, sex predicted the self-report of disability, not pain.

LBP is characterized by heterogeneous mechanisms of onset and pathoanatomic pain generators. Additionally, multiple studies have indicated that identification of pathoanatomy is difficult because of the many conditions that are also present in asymptomatic participants [57-63]. Development of an exercise-induced model of LBP is essential therefore to better control experimental investigations related to management of LBP. We believe that DOMS models will be useful for future studies of LBP. With these models we can test participants when they are pain free and examine factors that contribute to their pain experience after induction of LBP. In addition, we have the potential to establish comparison groups in whom the mechanism of onset is consistent resulting in the ability to use true experimental design in studies of LBP potentially allowing us to test responses to intervention in a homogenous LBP model. Also, there were participants who reported that they experienced no pain in the back or legs after performing the exercise protocol. Consequently, this presents the opportunity to study potential factors that might be protective of experiencing DOMS. This finding suggests additional relevance of our model because not all individuals who experience the same stress or stimulus go on to develop and complain of LBP. This is not possible in clinical studies and remains a novel part of this current study.

Models of endogenous muscle pain are particularly useful because they mimic the most common form of chronic pain, musculoskeletal pain [6,64]. Other endogenous experimental models of LBP have been recently presented that use injection of saline into spinal ligaments [65] and mulitifidus muscles [66]. Models using DOMS, such as presented in this current study and others [17-20], may have more clinical validity than these injection models given that DOMS is non-invasive and the course of symptoms lasts for days rather than hours or minutes potentially mimicking LBP for which an individual might seek intervention more closely. There was overlap between reports of pain of the highest 50% of participants with DOMS in our study (16 to 68) and the lowest 50% of the patients seeking intervention (0 to 50) [26] suggesting that the model may have the best comparison to patients with lower levels of pain and disability.

One important consideration, however, is that pain related to DOMS is likely to be of shorter duration than clinical pain. Additionally, pain was generated in this study in young healthy participants. Given the limited age range of participants in our study, future work will examine the development of pain in older adults to improve the external validity of DOMS as a model of LBP for which a patient might seek intervention. Other factors, such as catastrophizing, will need to be included in follow-up studies to better understand psychological influence and other work suggests an interaction between genetic and psychological factors contributes to pain and disability in the upper extremity [67]. Another limitation to consider is that we assume that the exercise protocol is the cause of the reported of pain intensity and disability at 24 and 48 hours. Without a group of participants performing sham exercise or in a control group we cannot be completely certain that the results of our study are attributable to participation in exercise.

## Conclusions

None the less, our results suggest overlap with clinical LBP for self-reports of pain as well as demonstrating association between pain and disability as would be expected in clinical pain. This model therefore may be most applicable to younger adults with lower levels of pain and disability. This work also supports the concept of multiple factors interacting to explain the development of pain and disability in LBP; however larger samples will be needed to develop a complete model.

## Competing interests

The authors declare that they have no competing interests.

## Authors' contributions

MDB conceived, and participated in the design of, the study; procured funding; participated in data collection; performed and interpreted statistical analyses; and helped to draft the manuscript. MEH participated in data collection and helped to draft the manuscript. SZG conceived, and participated in the design of, the study; interpreted statistical analysis; and helped to draft the manuscript. MER conceived, and participated in the design of, the study.

All authors read and approved the final manuscript.

## Pre-publication history

The pre-publication history for this paper can be accessed here:

http://www.biomedcentral.com/1471-2474/12/35/prepub
